# Changes in Degree Centrality of Network Nodes in Different Frequency Bands in Parkinson’s Disease With Depression and Without Depression

**DOI:** 10.3389/fnins.2021.638554

**Published:** 2021-03-22

**Authors:** Haiyan Liao, Jinyao Yi, Sainan Cai, Qin Shen, Qinru Liu, Lin Zhang, Junli Li, Zhenni Mao, Tianyu Wang, Yuheng Zi, Min Wang, Siyu Liu, Jun Liu, Chunyu Wang, Xiongzhao Zhu, Changlian Tan

**Affiliations:** ^1^Department of Radiology, The Second Xiangya Hospital, Central South University, Changsha, China; ^2^Medical Psychological Center, The Second Xiangya Hospital, Central South University, Changsha, China; ^3^Department of Neurology, The Second Xiangya Hospital, Central South University, Changsha, China

**Keywords:** Parkinson’s disease, depression, degree centrality, resting state functional magnetic resonance, frequency specificity

## Abstract

**Background:**

Depression induces an early onset of Parkinson’s disease (PD), aggravates dyskinesia and cognitive impairment, and accelerates disease progression. However, it is very difficult to identify and diagnose PD with depression (PDD) in the early clinical stage. Few studies have suggested that the changes in neural networks are associated with PDD, while degree centrality (DC) has been documented to be effective in detecting brain network changes.

**Objectives:**

The objectives of this study are to explore DC changes between patients with PDD and without depression (PDND) and to find the key brain hubs involved with depression in PD patients.

**Methods:**

One hundred and four PD patients and 54 healthy controls (HCs) underwent brain resting-state functional magnetic resonance imaging. The Data Processing and Analysis of Brain Imaging and Resting-State Functional Magnetic Resonance Data Analysis Toolkit were used for processing and statistical analysis. The DC value of each frequency band was calculated. One-way analysis of variance and a two-sample *t*-test for *post hoc* comparison were used to compare the differences of the DC values in different frequency bands among PDD, PDND, and healthy control group. Gaussian random field was used for multiple comparison correction. Pearson correlation analysis was performed between each individual’s DC map and clinical indicators.

**Results:**

The DC value of different brain regions changed in PDD and PDND in different frequency bands. The prefrontal lobe, limbic system, and basal ganglia were the main brain regions involved. PDD patients showed a wider range and more abnormal brain areas in the slow-4 frequency band (0.027–0.073 Hz) compared to the HCs. PDD showed a decreased DC value in the medial frontal gyrus, bilateral cuneus gyrus, right lingual gyrus, bilateral supplementary motor area (SMA), bilateral superior frontal gyrus, and left paracentral lobule, but an increased DC value in the bilateral brainstem, midbrain, bilateral parahippocampal gyrus, cerebellum, left superior temporal gyrus, bilateral insula, left fusiform gyrus, and left caudate nucleus in the traditional frequency band (0.01–0.08 Hz) compared to PDND patients. PDND patients displayed more abnormal functions in the basal ganglia in the slow-4 frequency band.

**Conclusion:**

The DC changes in PDD and PDND are frequency dependent and frequency specific. The medial frontal gyrus, SMA, and limbic system may be the key hubs for depression in PD.

## Introduction

Parkinson’s disease (PD) is one of the most common neurodegenerative diseases, and its incidence is only second to Alzheimer’s disease (Aaseth et al., 2018). Epidemiological studies demonstrated that the prevalence of PD in China is about 1.7% in the population of over 65 years of age (Liu and Chan, 2016), with a total number of patients being over 2 million. With the aging of the population, the prevalence of PD will continue to increase. A study by Lee and Gilbert (2016) estimated that the number of PD in China will exceed 5 million in 2020, and over half of all PD patients in the world will be Chinese by 2030. It will be a serious adversity on the health of the elderly and a heavy burden to families and society (Sagna et al., 2014).

The clinical symptoms of PD mainly include motor symptoms and non-motor symptoms with the diagnosis and treatment being mainly focused on the motor symptoms. Non-motor symptoms have been neglected, and only few studies have been conducted. Studies have found that the non-motor symptoms of PD patients often precede the motor symptoms by several years or even decades (Haehner et al., 2007). Some non-motor symptoms are expected to be the basis for early diagnosis of PD (Iranzo et al., 2013). Depression is the most common non-motor symptom of PD; it has an incidence of 30–40%, and it easily recurs (Aarsland et al., 2011). Depression seriously affects the quality of life in PD patients and is also an important factor in reducing the age of PD onset, aggravating dyskinesia and cognitive impairment, and accelerating disease progression (Yapici Eser et al., 2017). Currently, Parkinson’s disease with depression (PDD) is identified and diagnosed with behavioral observations and neuropsychological measurements, but it is very difficult to obtain an accurate diagnosis in the early clinical stage. Thus, it is significant to find new diagnostic markers for PDD.

With the advancement in neuroimaging technology, the use of new technologies to explore the neurobiological mechanism and to find objective diagnostic markers for PDD has become a research hotspot. It has been evident that resting-state functional magnetic resonance imaging (RS-fMRI) is a feasible method for exploring the pathogenesis and pathophysiological changes of PDD (Wen et al., 2016; Thobois et al., 2017). At present, data analysis methods based on resting state are also emerging one by one, mainly studying human brain function from functional differentiation and functional integration. In contrast; only a few studies have explored the roles of the neural networks in PDD. One study using the amplitude of low-frequency fluctuation (ALFF) and regional homogeneity (ReHo) analysis methods demonstrated that the regional brain function changes in the prefrontal-limbic system, basal ganglia, and default-mode network are related to PD with depression (PDD; Wen et al., 2013; Sheng et al., 2014; Hu et al., 2015a,b). A study by Hu et al. (2015a,b) using seed-based functional connectivity (FC) analysis suggested that the dysfunction of the prefrontal-limbic loop may be an important mechanism of PD depression. However, it is not clear whether the disease damages certain neural networks other than the prefrontal-limbic loop. A study based on whole-brain FC analysis found that the dorsolateral prefrontal cortex, superior temporal gyrus, and posterior cingulate gyrus are key nodes in the brain network structure (Lou et al., 2015). Therefore, exploring the neural mechanism of PD associating with depression at the system or network level will help us better understand the neural model of the disease.

Although previous RS-fMRI studies have used the ALFF, ReHo analysis, and FC analysis to investigate alterations in regional brain activity and default mode networks in depressed PD patients (Wen et al., 2013; Luo et al., 2014; Sheng et al., 2014; Hu et al., 2015a; Liang et al., 2016), degree centrality (DC) is a graph theoretical analysis based on voxel level, which can describe the importance of nodes in the network. It is used in the analysis of brain function networks to explore the nodes of brain information transmission. DC does not need to select a region of interest and at the same time has a high repeatability (Zuo and Xing, 2014); it can provide valuable information on the changes of nodes in the human brain connection caused by diseases (Di Martino et al., 2013). At present, DC has been widely used in exploring the neurobiological mechanism of brain network changes in various diseases (Shen et al., 2015; Guo et al., 2016) but rare in PDD.

Zuo et al. (2010) has divided the low-frequency band into four sub-frequency bands: slow-5 band (0.01–0.027 Hz), slow-4 band (0.027–0.073 Hz), slow-3 band (0.073–0.198 Hz), and slow-2 band (0.198–0.25 Hz). Traditional RS-fMRI studies believe that the cerebral blood oxygen level-dependent signals (BOLD) in the 0.01–0.08 Hz frequency band are physiologically significant (Biswal et al., 1995). Conversely, other frequency bands are considered noises or interferences. Specific sub-band analysis can avoid the interference of physiological noise that is active in other frequency bands, thereby improving the accuracy of detecting abnormal brain regions and enhancing the ability to detect detailed information and spatial distribution of brain regions (Wang et al., 2014). Although some studies reported the influence of different frequency bands on the global characteristics of the whole-brain functional network and brain state (Baliki et al., 2011), there is no DC study on PDD with different frequency bands. In this study, the signals in the two frequency bands of slow-3 and slow-2 are discarded because signals in these two frequency bands mainly reflect the white matter signal and high-frequency physiological noise, but the signal vibrations in the slow-5 and slow-4 band are closely related to the BOLD signal of brain gray matter. Thus, slow-5 and slow-4 bands are suitable for correlation analysis between brain dysfunction and functional processing (Salvador et al., 2008; Zuo et al., 2010; Wang et al., 2020). We therefore hypothesized that exploring DC changes within slow-5 and slow-4 bands could find the key brain hubs involved with depression in PD patients.

This study used the DC method with the following sub-frequency bands: traditional, slow-5, and slow-4 frequency band to study the characteristics of node attributes in brain networks in PDD and PDND patients. Our study will find the key brain hubs of PDD, clarify its spectrum-specific changes, and propose whether sub-frequency DC changes can distinguish PDD from PDND; this will provide a new research method for the potential pathogenesis of PDD.

## Materials and Methods

### Research Objects

This study was approved by the Ethics Committee of the Second Xiangya Hospital of Central South University. One hundred and forty-seven patients with PD were recruited from the Neurology Clinic of the Second Xiangya Hospital from December 2015 to December 2019. A total of 147 PD patients were enrolled in this study. 10 patients with other intracranial lesions, eight patients who were unable to complete the medical history collection and questionnaire assessment, 10 patients with dementia, and 10 patients with a history of depression before suffering from PD were excluded. Five patients were excluded due to data processing failure, and 104 patients were finally included into analysis, including 40 PDD and 64 PDND patients. At the same time, 54 age-, and gender-similar healthy controls (HCs) were recruited from the community. All participants in the study signed a written informed consent.

### Inclusion and Exclusion Criteria

Parkinson’s disease with depression and PDND inclusion criteria were as follows: (1) patient met the diagnostic criteria of PD in the brain bank of the British PD Association; (2) patient had no obvious cognitive impairment (Mini-Mental State Examination, MMSE, illiteracy > 17 scores, elementary school > 20 scores, and junior high school and above > 24 scores); (3) patient was right-handed; (4) patient with PDD met the diagnostic criteria for depression in the fifth edition of the Diagnostic and Statistical Manual of Mental Disorders (DSM-V), meaning that PDD patients have at least one major depressive symptom (depressed mood or loss of interest or pleasure), which lasted more than 2 weeks. The 17-item Hamilton Depression Scale (Hamilton Depression Scale-17, HAMD-17) was used to assess the severity of depression. When the score was ≥ 7 points, it was considered depression; and (5) all anti-PD drugs were stopped for at least 12 h before clinical evaluation and MRI scans.

Exclusion criteria for PDD and PDND were as follows: patients with Parkinson’s syndrome or Parkinson’s superimposed syndrome being caused by other diseases; patients with a history of stroke, depression, dementia, or other central nervous system diseases; patients with contraindications to magnetic resonance examination; and patients who had excessive head movement during the process affecting data analysis.

The inclusion criteria for healthy control group were as follows: (1) no history of neuropsychiatric diseases or major organic diseases; (2) age, gender, and education matched with the PD patient; (3) right-handed; (4) no obvious cognitive impairment; and (5) had an understanding of the research content and had the willingness to participate in/ability to complete the entire experiment.

The exclusion criteria of HCs were as follows: (1) those who were contraindicated for MRI examinations; (2) those who had a family history of mental illness; (3) those who had a history of long-term alcohol abuse or other drug abuse; and (4) those who had excessive head movement during the examination, which affects data analysis.

### Clinical and Neuropsychological Evaluations

All clinical evaluations and neuropsychological determinations were performed during the off-period (12 h after the patient stopped taking drugs for PD). Two neurologists and psychologists collected and recorded the age, gender, medical history, and history of disease course of each PDD patient, PDND patient, and healthy control; the Hoehn–Yahr classification of PD patients was determined based on neurological examinations. All PD patients completed the Unified Parkinson’s Disease Rating Scale (UPDRS), Beck Depression Inventory-21 (BDI-21), Hamilton Depression Scale-17 (HAMD-17), and the MMSE Scale. The third part of the Unified Parkinson’s Disease Rating Scale (UPDRS-III) and the Hoehn–Yahr grading scale were used for the classification of motor disability and PD. The Mini cognitive scale (Mini-Cog) was used to assess the cognitive function. The Beck Depression Inventory (BDI-21) and Hamilton Depression Inventory (HAMD-17) were used to assess the individual’s depressive state.

### Magnetic Resonance Imaging Data Acquisition

All MRI data were collected by the 3T Siemens Skyra magnetic resonance imaging instrument in the Radiology Department of the Second Xiangya Hospital. Before scanning, the operator explained the scanning requirements to the subjects. Participants were asked to lie down in a supine position in a magnetic resonance machine equipped with a standard head coil and then asked to close their eyes and relax but not fall asleep. The subjects were closely observed whether they fell asleep during the scan. After the scan, the subjects were also asked if they were sleeping. The subjects who were sleeping were excluded from data analysis. All subjects’ heads were fixed with sponges, and earphones were worn to reduce noise.

T1-weighted structure image data and resting-state image data of subjects were collected during the scanning process. The resting-state data were obtained by using echoplanar imaging (EPI) sequence. The specific parameters were as follows: repetition time (TR) = 2,500 ms, echo time (TE) = 25 ms, slice = 39, slice thickness = 3.5 mm, gap = 0 mm, voxel size = 3.8 mm × 3.8 mm × 3.5 mm, flip angle = 90°, field of view (FOV) = 240 mm, matrix = 64 × 64, and scanning to obtain image data at 200 time points (volume = 200). The whole scan procedure lasted for 508 s.

The T1-weighted structure image data were acquired by 3D magnetization-prepared rapid gradient-echo (MPRAGE) sequence. The specific parameters were as follows: TR = 1,900 ms, TE = 2.01 ms, slice = 176, slice thickness = 1.0 mm, voxel size = 1.0 mm × 1.0 mm × 1.0 mm, flip angle = 9°, FOV = 256 mm, and matrix = 256 × 256.

### Imaging Data Preprocessing

On the MatLab2012a platform, the MRI resting-state data processing software Data Processing and Analysis of Brain Imaging (DPABI_V3.1_180801) and the Resting-State Functional Magnetic Resonance Data Analysis Toolkit (REST) were used for data preprocessing. The main steps included (1) converting the data in DICOM format to NIFTI format; (2) removing the initial 10 slices, (3) correcting the time layer to align the scanning time of all slices to the reference slice; (4) excluding subjects whose head movement exceeds 3 mm or rotates more than 3° (Yan et al., 2013; Wang et al., 2020); (5) performing spatial standardization: the standard brain analysis was performed based on the T1-weighted structure phase and the Montreal Neurological Institute (MNI) functions as template space standardization; (6) removing linear drift; (7) removing covariates (24 head movement parameters, white matter signals, and cerebrospinal fluid signals); and (8) using low-frequency filtering: a bandpass filter (BPF) was used to extract signals in the classic frequency band (0.01–0.08 Hz), slow-5 sub-band (0.01–0.027 Hz), and slow-4 sub-band (0.027–0.073 Hz) to remove interference from high-frequency and low-frequency signals.

### Degree Centrality Analysis

The DC value of each frequency band including the traditional frequency band, the slow-5 sub-band, and the slow-4 sub-band was calculated. Briefly, individual Pearson’s correlation coefficients were computed in a prior probability brain gray matter mask in SPM8 between the time course of a given voxel and all other whole-brain voxels within the template. The binarized DC value of the whole brain at the voxel level was first calculated with the correlation threshold being set at *r* ≥ 0.25, and then, a whole-brain FC matrix for each subject was generated. The obtained individualized DC value was then converted to a Fisher *Z* value to conform the normal distribution and then get the *Z*-valued DC distribution map of each subject; finally, the obtained DC map was smoothed with a Gaussian kernel whose full width at half maximum was 4 mm isotropic for statistics analysis (Hu et al., 2017).

### Statistical Analysis

One-way analysis of variance (ANOVA; one tail) and a two-sample *t*-test for *post hoc* comparison (two tail) were used to compare the differences of the DC values in different frequency bands among the three groups (PDD, PDND, and HCs); the threshold level was set at *p* < 0.05. Gaussian random field (GRF) was used for multiple comparison correction. First, ANOVA was performed in PDD, PDND, and HC groups to obtain the F map with a GRF correction (voxel level *p* < 0.05, cluster level *p* < 0.05). Second, the brain areas with differences among the three groups were used as a mask, and the two-sample *t*-test was then performed for pairwise comparisons in this mask (GRF, voxel level *p* < 0.005, and cluster level *p* < 0.05). In order to clarify which brain area in PD patients with depression and without depression was related to clinical indicators (disease course, depressive symptoms, and motor symptoms), voxel-based Pearson correlation was carried out between the DC diagram of each individual and related clinical indicators; a *p* < 0.05 (uncorrected) indicated that the difference was statistically significant. Age, gender, years of education, gray matter volume, and MMSE score have been used as covariates in the above analysis.

## Results

### Comparison of Demographic and Clinical Variables

The comparisons of demographic and clinical variables are listed in Table 1. There were no significant differences in age, gender, education level, MMSE score, relative gray matter volume, relative white matter volume, and mean power framewise displacement (FD) between the three groups. The relative gray matter volume and relative white matter volume used in the data analysis were measured from the segmented T1 images with CAT12 (A Computational Anatomy Toolbox for SPM 12). There were no significant differences between PDD and PDND patients in the course of disease, Hoehn & Yahr (H&Y) classification, and UPDRS-III scores. The HAMD and BDI scores were significantly higher in PDD patients than in PDND patients and HCs (*p* < 0.001).

**TABLE 1:** Demographic and clinical characteristics of the participants (mean ± SD).

Variable	HC (*N* = 54)	PDD (*N* = 40)	PDND (*N* = 64)	*P*
Gender (M/F)	25/29	19/21	35/29	0.619^a^
Age (years)	56.446 ± 8.564	56.250 ± 8.133	56.950 ± 9.967	0.920^b^
Education (years)	8.027 ± 3.560	6.650 ± 2.966	7.367 ± 3.390	0.141^b^
Disease duration (years)	NA	2.600 ± 2.481	2.304 ± 1.747	0.174^c^
MMSE	26.732 ± 3.250	26.125 ± 2.866	27.067 ± 2.469	0.277^b^
HAMD-17	2.289 ± 2.615	14.639 ± 4.859	3.283 ± 2.116	<0.001^b^
BDI-21	9.804 ± 9.353	23.000 ± 10.570	8.733 ± 5.862	<0.001^b^
UPDRS-III	NA	19.550 ± 12.543	14.800 ± 9.896	0.148^c^
HY	NA	1.650 ± 0.718	1.550 ± 0.642	0.167^c^
REL-GMV	0.410 ± 0.025	0.408 ± 0.025	0.405 ± 0.024	0.686^b^
REL-WMV	0.400 ± 0.023	0.336 ± 0.021	0.333 ± 0.025	0.727^b^
Mean power FD	0.098 ± 0.051	0.084 ± 0.037	0.091 ± 0.042	0.078^b^

*PDD, Parkinson’s disease with depression; PDND, PD patients without depression; HC, healthy control; HAMD-17, 17-item Hamilton Depression Rating Scale; BDI-21, 21-item Beck’s Depression Inventory; UPDRS-III, Unified Parkinson’s Disease Rating Scale-motor part III; H&Y, Hoehn & Yahr staging scale; MMSE, Mini-Mental State Examination; REL-GMV, relative gray matter volume; REL-WMV, relative white matter volume; FD, framewise displacement; and NA, not applicable.*

*For comparisons of demographics:*

*^*a*^*p* value for the gender difference was obtained by chi-square test.*

*^*b*^*p* values were obtained by one-way analysis of variance (ANOVA) tests.*

*^*c*^*p* values were obtained by two-sample *t*-test.*

### DC Difference in the Traditional Frequency Band (0.01–0.08 Hz) Between PDD, PDND, and HC Group (ANOVA Result)

**FIGURE 1:**
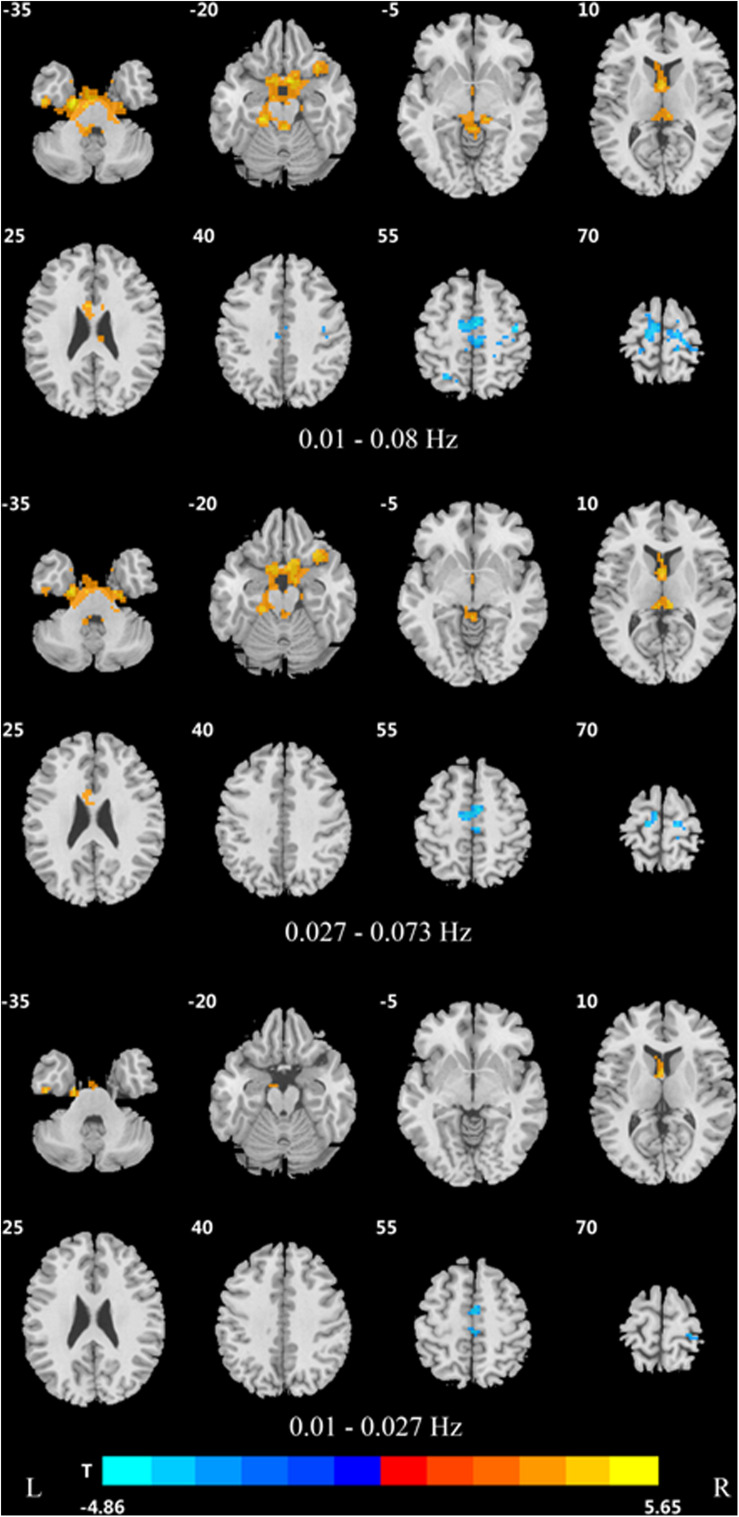
Differences in DC of traditional frequency bands (0.01–0.08 Hz) between PDD, PDND, and healthy control group (ANOVA result, GRF correction, voxel *p* < 0.05, cluster level *p* < 0.05, and one tailed). DC, degree centrality; PDD, Parkinson’s disease with depression; PDND, Parkinson’s disease without depression; ANOVA, analysis of variance; and GRF, Gaussian random field.

Significant DC differences were observed in the left calcarine gyrus, left cuneus gyrus, left precuneus, medial frontal gyrus, precentral gyrus, and Brodmann area 6 among the three groups (Figure 1).

**FIGURE 2:**
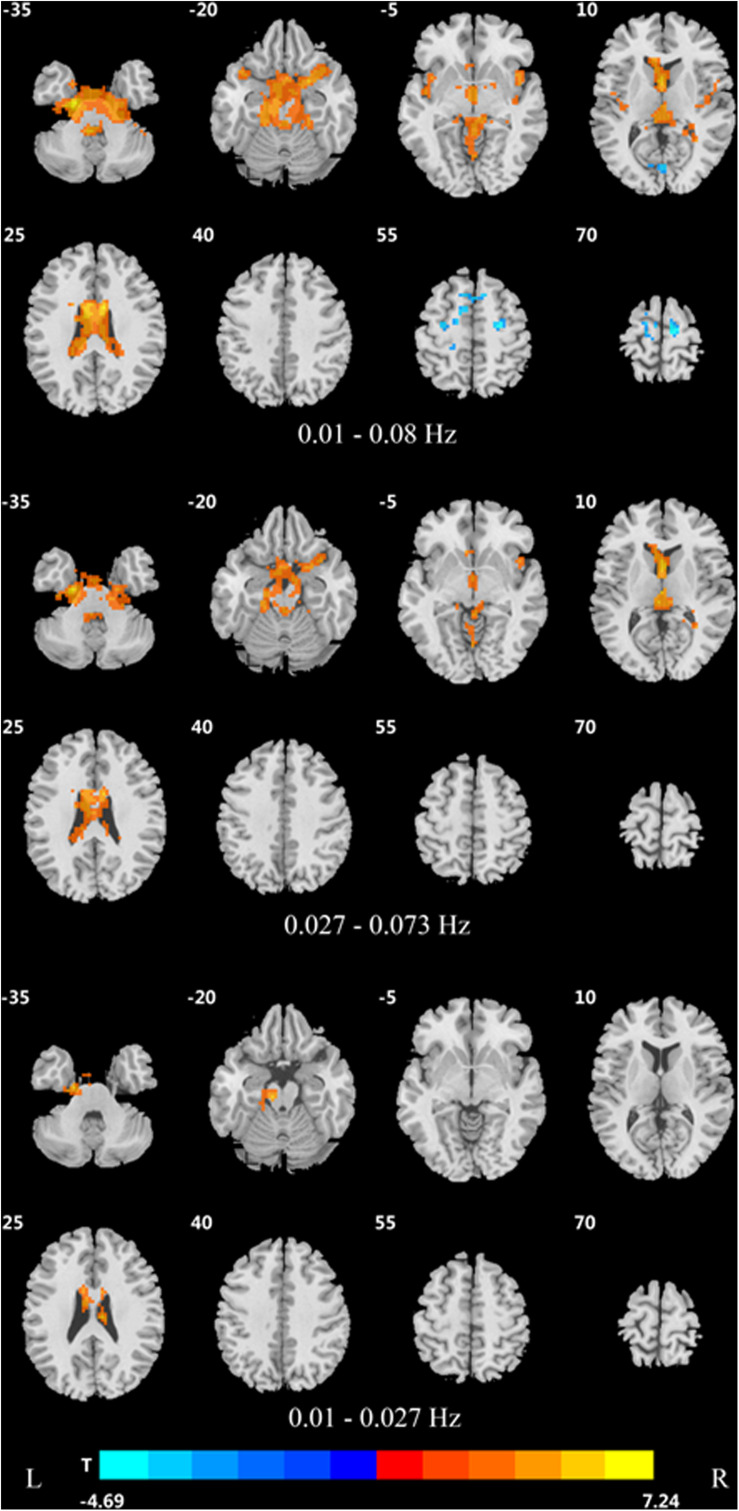
Difference in DC values of three different frequency bands between PDD and healthy controls (*post hoc t*-test, GRF, voxel level *p* < 0.005, cluster level *p* < 0.05, and two tailed).

**FIGURE 3:**
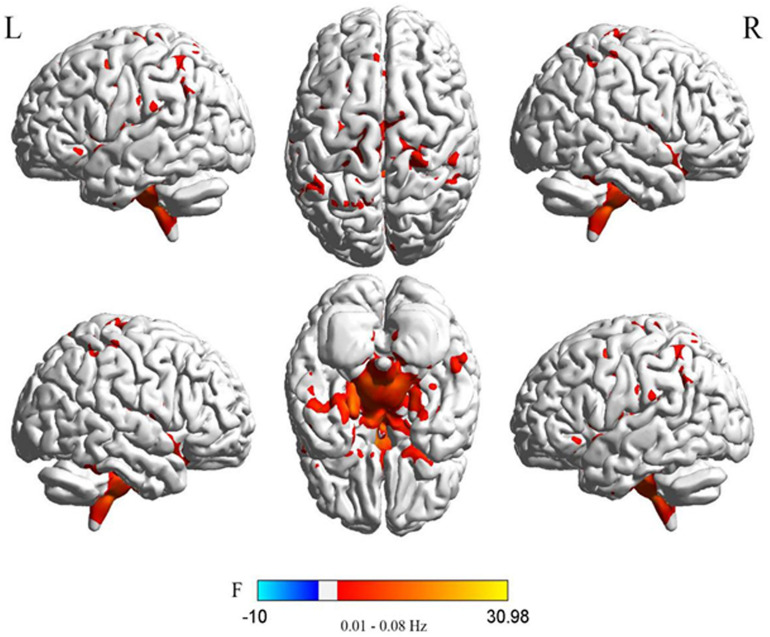
Difference in DC values of three different frequency bands between the two PDD and PDND groups (*post hoc t*-test, GRF, voxel level *p* < 0.005, cluster level *p* < 0.05, and two tailed).

**FIGURE 4:**
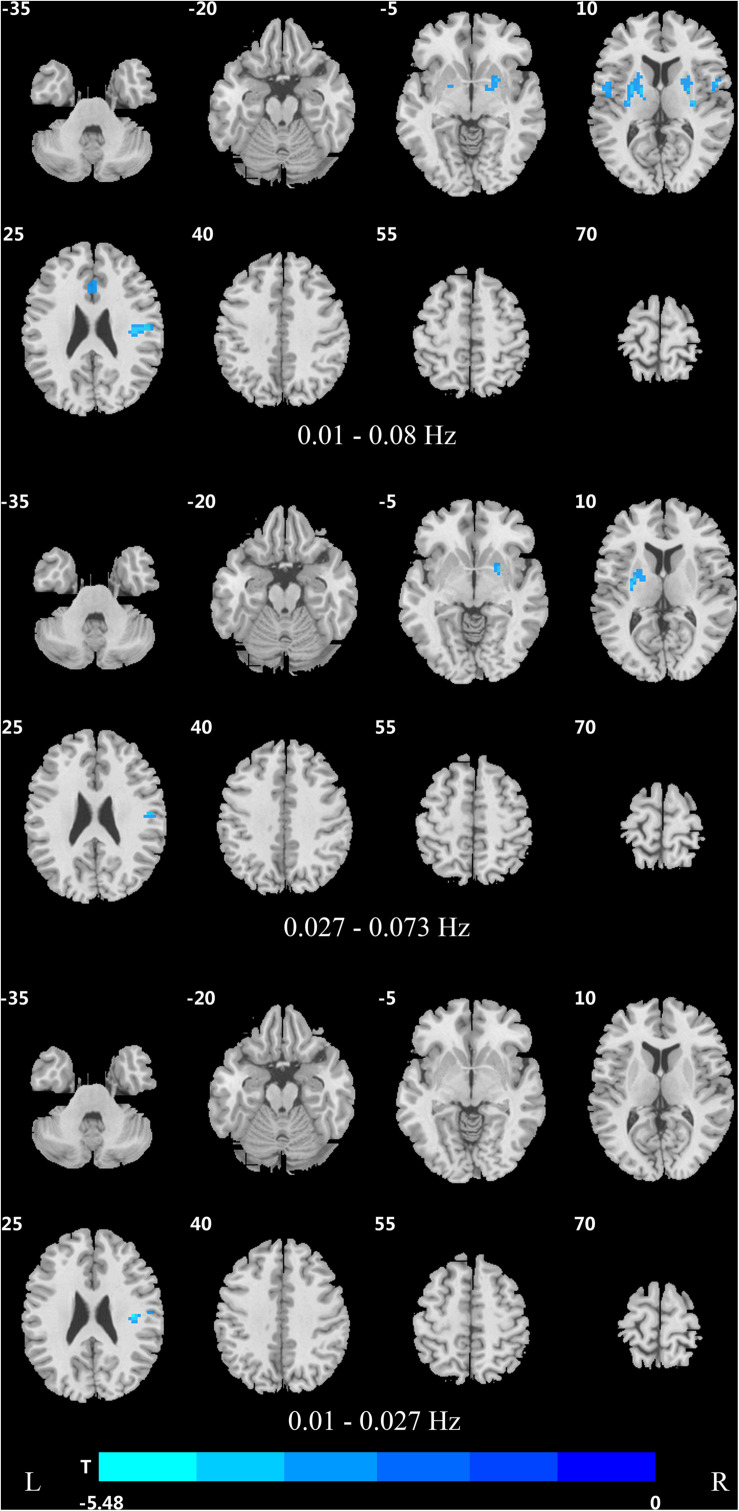
Difference in DC values of three frequency bands between PDND and healthy control group (*post hoc t*-test, GRF, voxel level *p* < 0.005, cluster level *p* < 0.05, and two tailed).

In the traditional frequency band, the DC value in the medial frontal gyrus, bilateral supplementary motor area (SMA), bilateral precentral gyrus, bilateral postcentral gyrus, bilateral paracentral lobules, and left superior parietal gyrus was decreased in PDD patients compared to the HCs. In contrast, the DC value was increased in PDD patients in the bilateral brainstem, midbrain, bilateral parahippocampal gyrus, cerebellum, superior temporal gyrus, and left fusiform gyrus compared to the HCs (Figure 2 and Table 2). The DC value was decreased in the medial frontal gyrus, bilateral cuneus gyrus, right lingual gyrus, bilateral SMAs, bilateral superior frontal gyrus, and the left paracentral lobules, but increased in the bilateral brainstem, midbrain, bilateral parahippocampal gyrus, cerebellum, left superior temporal gyrus, bilateral insula, left fusiform gyrus, and the left caudate nucleus in PDD patients compared to PDND patients (Figure 3 and Table 3). The DC value was decreased in the bilateral putamen, bilateral Rolandic operculum, left postcentral gyrus, left anterior cingulum gyrus, and left cingulum mid gyrus in PDND patients compared to HCs (Figure 4 and Table 4).

**TABLE 2:** Differences of DC values in three frequency bands between PDD and healthy control group (GRF, voxel level *p* < 0.005, cluster level *p* < 0.05, and two tailed).

Brain area	Cluster	Brodmann area	Voxel size	MNI coordinates at peak	*T* value at peak
**DC decreased brain area**					
**Traditional frequency band (0.01–0.08 Hz)**					
Medial frontal gyrus/supp_motor_area_R/L (aal)/precentral_R/L (aal)/paracentral_lobule_L/R (aal)/postcentral_R/L (aal)	2	6, 4, 3, 24	644	−6 −12 60	−4.8627
Parietal_sup_L (aal)	3	7	59	−24 −60 54	−3.8203
**Slow-5 frequency band (0.01–0.027 Hz)**					
Medial frontal gyrus/supp_motor_area_R (aal)	6	6	46	3 −6 54	−3.9466
Postcentral_R (aal)	7	3, 4, 1	29	27 −30 72	−3.7868
**Slow-4 frequency band (0.027–0.073 Hz)**					
Medial frontal gyrus/supp_motor_area_L (aal)/paracentral_lobule_L (aal)	3	6, 24, 32, 31	180	−6 −12 66	−4.4377
Postcentral_R (aal)	4	4, 6, 3	45	24 −30 60	−3.813
**DC increased**					
**Traditional frequency band (0.01–0.08 Hz)**					
Left/right brainstem/midbrain/parahippocampal_L/R (aal)/cerebellum_4_5_L (aal)/superior temporal gyrus/temporal_pole_sup_L (aal)/fusiform_L	1	28, 38, 20, 47, 34, 35, 11, 36	2799	−18 −15 −36	5.6501
**Slow-5 frequency band (0.01–0.027 Hz)**					
Right/left brainstem	1		35	9 −12 −51	3.8683
Parahippocampal_L (aal)	2	20	120	−27 −6 −48	5.6285
Temporal_Inf_L (aal)	3	20	28	−51 −21 −42	4.913
Temporal_pole_sup_L (aal)	4	11, 47	78	−12 21 −30	4.1271
Caudate_L (aal)	5		32	−3 3 9	4.2003
**Slow-4 frequency band (0.027–0.073 Hz)**					
Parahippocampal_L/R (aal)/cerebellum_4_5_L (aal)/temporal_pole_sup_L/R (aal)/frontal_inf_orb_R (aal)/fusiform_L (aal)/insula_R	1	38, 28, 47, 34, 35, 11, 36, 33	2,009	−24 0 −51	5.6186
Temporal_Inf_L (aal)	2	20	49	−39 −3 −51	3.9262

**TABLE 3:** Differences in DC values of three frequency bands between PDD and PDND group (GRF correction, voxel level *p* < 0.005, cluster level *p* < 0.05, and two tailed).

Brain area	Cluster	Brodmann area	Voxel size	MNI coordinates at peak	*T* value at peak
**DC decreased**					
**Traditional frequency band (0.01–0.08 Hz)**					
Calcarine_L/R (aal)/lingual_R (aal)	3	18, 23, 17	62	12 −81 0	−4.0376
Supp_motor_area_L (aal)/Paracentral_Lobule_L (aal)	4	6, 4, 3	167	−18 −9 63	−4.686
Supp_motor_area_R/L (aal)/frontal_sup_R/L (aal)/medial frontal gyrus	5	6, 8, 32	81	15 9 60	−4.2856
Supp_motor_area_R (aal)/medial frontal gyrus	6	6	92	12 −15 69	−4.5108
**Slow-5 frequency band (0.01–0.027 Hz) No**					
**Slow-4 frequency band (0.027–0.073 Hz) No**					
**DC increased**					
**Traditional frequency band (0.01–0.08 Hz)**					
Left/right brainstem/midbrain/parahippocampal_L/R (aal)/insula_R (aal)/cerebellum_4_5_L (aal)/fusiform_L (aal)/caudate_L (aal)	1	28, 13, 38, 34, 24, 20, 35, 36, 22, 30, 33, 25	4,512	−21 −15 −36	7.2402
Superior temporal gyrus/temporal_pole_sup_L (aal)/insula_L (aal)	2	13, 38, 2,247, 6, 21	194	−33 15 −27	4.4345
**Slow-5 frequency band (0.01–0.027 Hz)**					
Left brainstem/midbrain/parahippocampal_L (aal)/cerebellum_4_5_L (aal)/fusiform_L (aal)	1	28	218	−18 −15 −39	6.1376
Cingulate gyrus	2	33	110	9 −18 24	4.8639
**Slow-4 frequency band (0.027–0.073 Hz)**					
Left/right brainstem/midbrain/parahippocampal_L/R (aal)/cerebellum_4_5_L (aal)/insula_R (aal)/fusiform_L (aal)/caudate_L (aal)	1	28, 47, 38, 24, 34, 35, 20, 22, 25, 33, 13	2,748	−21 −15 −36	6.3712

**TABLE 4:** Difference of DC values in three frequency bands between PDND group and healthy control group (GRF correction, voxel level *p* < 0.005, cluster level *p* < 0.05, and two tailed).

Brain area	Cluster	Brodmann area	Voxel size	MNI coordinates at peak	*T* value at peak
**DC decreased**					
**Traditional frequency band (0.01–0.08 Hz)**					
Putamen_R (aal)/Rolandic_oper_R (aal)	1	13, 43, 6	199	54 −12 21	−5.4838
Putamen_L (aal)	2		105	−27 −3 9	−4.405
Rolandic_oper_L (aal)/postcentral_L (aal)	3	43, 6	82	−45 0 18	−4.6693
Cingulum_ant_L (aal)/cingulum_mid_L (aal)	4	24, 32	54	−3 15 33	−4.0495
**Slow-5 frequency band (0.01–0.027 Hz)**					
Rolandic_oper_R (aal)	1	43, 13	38	39 −15 24	−4.8727
**Slow-4 frequency band (0.027–0.073 Hz)**					
Putamen_R (aal)	1		13	24 6 −6	−4.2337
Putamen_R (aal)	2		9	27 −6 3	−3.3092
Putamen_L (aal)	3		39	−27 −3 6	−4.0764
Rolandic_oper_R (aal)	4		13	54 −12 21	−5.2369

In the slow-4 frequency band, the DC value was lowered in the medial frontal gyrus, left SMA, left paracentral lobule, and right postcentral gyrus, but increased in the bilateral parahippocampal gyrus, cerebellum, bilateral superior temporal gyrus, right orbit inferior frontal gyrus, left fusiform gyrus, right insula, and left inferior temporal gyrus in PDD patients compared to HCs (Figure 2 and Table 2). The DC value was increased in the bilateral brainstem, midbrain, bilateral parahippocampal gyrus, cerebellum, left fusiform gyrus, right insula, and left caudate nucleus in PDD patients compared to PDND patients (Figure 3 and Table 3). The DC value was reduced in the bilateral lenticular putamen and the right Rolandic operculum in PDND patients when compared to HCs (Figure 4 and Table 4).

In the slow-5 frequency band, the DC value was decreased in the medial frontal gyrus, the right SMA, the postcentral gyrus, the bilateral brainstem, and the left parahippocampal gyrus, but increased in the inferior temporal gyrus, the left superior temporal gyrus, and the left caudate nucleus in PDD patients compared to HCs (Figure 2 and Table 2). The DC value was increased in the left brainstem, midbrain, left parahippocampal gyrus, left cerebellum, left superior temporal gyrus, and right cingulate gyrus in PDD patients compared to PDND patients (Figure 3 and Table 3). The DC value was reduced in the right Rolandic operculum in PDND patients compared to HCs (Figure 4 and Table 4).

### The Correlation Analysis of DC Values and Clinical Indicators in the PDD and PDND Patients in the Three Frequency Bands

Correlation analysis was performed between the DC value of each PD patient in the three frequency bands and the course of disease, depression score (HAMD-17 and BDI-21), motor symptom score (UPDRS-III), H&Y classification, and MMSE (Table 5).

**TABLE 5:** Correlation between changes of DC value and clinical indicators in PDD and PDND groups in three frequency bands (*p* < 0.05, uncorrected).

Frequency band	Group	Brain area	Clinical index	Correlation coefficient
Traditional frequency band (0.01–0.08 Hz)	PDD-PDND (PDD)	Cluster 1 (left/right brainstem/midbrain/parahippocampal_L/R)	BDI-21	*r* = 0.350, *p* = 0.036
Traditional frequency band (0.01–0.08 Hz)	PDD-PDND (PDD)	Cluster 2 (temporal_pole_sup_L)	BDI-21	*r* = 0.425, *p* = 0.010
Traditional frequency band (0.01–0.08 Hz)	PDND-HC (PDND)	Cluster 4 (cingulum_ant_L/cingulum_mid_L)	HAMD-17	*r* = -0.300, *p* = 0.022
Slow-4 frequency band (0.027–0.073 Hz)	PDD-PDND (PDD)	Cluster 1 (left/right brainstem/midbrain/parahippocampal_L/R)	BDI-21	*r* = 0.333, *p* = 0.047
Slow-4 frequency band (0.027–0.073 Hz)	PDD-HC (PDD)	Cluster 3 (medial frontal gyrus/supp_motor_area_L/paracentral_lobule_L)	UPDRS-III	*r* = 0.349, *p* = 0.038

In the traditional frequency band, the increased DC value in the cluster 1 brain areas (brainstem, midbrain, and bilateral parahippocampal gyrus) and cluster 2 brain areas (left superior temporal gyrus) of PDD and PDND patients positively correlated with BDI-21 scores, with a correlation coefficient of 0.350 and 0.425 (*p* = 0.036, 0.010, and uncorrected), respectively. The decreased DC value in cluster 4 brain areas (left anterior cingulum gyrus and cingulum mid gyrus) of PDND patients negatively correlated with HAMD-17 scores with a correlation coefficient of -0.300 (*p* = 0.022, uncorrected).

In the slow-4 frequency band, the increased DC value of PD patients in the cluster 1 brain areas (mainly brainstem, midbrain, and bilateral parahippocampal gyrus) positively correlated with the BDI-21 scores, with a coefficient of 0.333 (*p* = 0.047, uncorrected); the decreased DC value in the cluster 3 brain area (left SMA) positively correlated with the UPDRS-III score, with a correlation coefficient of 0.349 (*p* = 0.038, uncorrected).

In the slow-5 frequency band, the changes in the DC values showed no statistically significant correlation with the clinical indicators (Table 5).

## Discussion

This is the first study to explore the changes in the central nodes of the whole brain in PD patients with and without depression in both the time and space dimensions; such changes were studied using the DC and multi-band analysis methods at the voxel level. This study reported several novel findings.

First, this study found that DC changes in PDD and PDND patients are frequency dependent and frequency specific.

This study demonstrated that PDD patients exhibited an abnormal DC value in multiple frequency bands in the prefrontal lobe, limbic system, and basal ganglia; however, the abnormalities were wider in the slow-4 frequency band, suggesting that slow-4 band is more suitable for detecting DC abnormalities in the limbic system. At the same time, the DC value was increased or decreased in multiple brain areas in PDD patients compared to PDND patients in the traditional frequency band, but no brain area displayed a reduced DC value in the slow-5 and slow-4 frequency band. We speculate that the traditional frequency band is more sensitive to distinguish abnormal brain areas between PDD and PDND patients. In addition, changes of DC in PDND patients mainly occur in slow-4, indicating that slow-4 is more sensitive in detecting abnormal brain function activities related to PDND. Thus, the slow-4 frequency band can provide more in-depth diagnostic information for PDND patients than other frequency bands.

This study also revealed that PDD and PDND have a certain frequency dependence. PDD and PDND exhibited different abnormal patterns of brain function networks in different frequency bands, and the corresponding DC abnormal brain areas have frequency specificity. Some specific frequency bands are more sensitive in detecting brain function activities. Combining these different abnormal patterns can better distinguish PDD from PDND. The traditional frequency band is more sensitive to detecting abnormalities in the neural activity of the prefrontal lobe and basal ganglia. The slow-4 band is more sensitive to detecting abnormal neural activity in the bilateral parahippocampal gyrus and fusiform.

Previous studies also suggest that the functional connections of brain sub-networks have different frequency-specific characteristics. For example, a previous study found that the functional connections of some cortical networks are mainly concentrated on one ultra-low frequency range (0.01–0.06 Hz), while the functional connections of some edge networks are mainly distributed in a relatively wide frequency range (0.01–0.14 Hz); these indicate that the functional connections of the brain sub-networks have different frequency-specific characteristics (Wu et al., 2008). Studies have also broken down the fMRI oscillation frequency into different frequency bands and found that the brains of PD patients have specific oscillation frequency abnormalities (Zhang et al., 2013; Hou et al., 2014; Song et al., 2015).

Second, this study found that the medial frontal gyrus is a key hub for Parkinson’s depression.

In this study, we found that compared to HCs and PDND patients, the DC value of the medial frontal gyrus was decreased in multiple frequency bands in PDD patients, suggesting that the medial frontal gyrus is a key hub of Parkinson’s depression. Previous PET and SPECT studies have shown that decreases of local glucose metabolism and cerebral blood flow in the medial orbital gyrus and middle frontal gyrus are related to PDD (Mayberg et al., 1990; Kim et al., 2016). The studies using low-frequency amplitude (Luo et al., 2014) and local consistency (Sheng et al., 2014) as indicators also observed abnormalities in the local activity or functional connection of the prefrontal lobe in PDD patients. A meta-analysis has shown that repetitive transcranial magnetic stimulation of the prefrontal cortex has a significantly positive antidepressant effect in patients with PD (Zhou et al., 2019). In addition, Lou et al. (2015) found that the eigenvector centrality of the left superior frontal gyrus and middle frontal gyrus was abnormal in PD patients with depression.

Third, this study found that the SMA is another key hub in the pathogenesis of depressive PD.

The SMA includes two anatomically and functionally different areas: the pre-supplementary motor area (pre-SMA) and SMA (Lehéricy et al., 2004). The SMA has a wide range of functions, involving in sports, digital cognition, time and space processing, music and language processing, and working memory (Cona and Semenza, 2017). A significant decrease in the metabolism of the SMA is a characteristic of patients with refractory depression (Li et al., 2015). In this study, we found that when compared with HCs and PDND patients, PDD patients showed reduced DC values in SMA in multiple frequency bands. Additionally, in the slow-4 frequency band, the decreased DC value of SMA was positively correlated with the UPDRS-III score. Therefore, we believe that the decrease in DC value in SMA may be related to the occurrence of mood disorders and the rapid decline of exercise ability in PDD patients.

Fourth, this study found that the limbic system is an important node in the occurrence of depression in PD.

The parahippocampal gyrus, fusiform gyrus, anterior cingulate cortex, insula, and superior temporal gyrus are important components of the limbic system, and they play an important role in regulating cognition and emotion. Abnormal function of the anterior cingulate cortex can lead to cognitive impairment and affective disorders. The anterior cingulate cortex is another key hub in the pathogenesis of PDD (Wang et al., 2018). A study by Feldmann et al. (2008) using the voxel-based morphometry (VBM) method found that the gray matter volume in the left lower orbitofrontal gyrus, bilateral straight gyrus, and right superior temporal gyrus was decreased in PD patients with depression compared to PD patients without depression. Simultaneously, the gray matter volume in the right superior temporal gyrus was negatively correlated with the depression score (Feldmann et al., 2008). Ballanger et al. (2012) also found that the dysfunction of serotonin receptors in the limbic system is involved in the production and progress of Parkinson’s depressive symptoms. In this study, we found that PDD patients have a wide range of increased DC values in the limbic system in multiple frequency bands compared to PDND patients and normal controls. In the traditional frequency band, the increased DC values in cluster 1 and cluster 2 brain areas positively correlated with the BDI-21 score, and the decreased DC value of the cingulate gyrus in cluster 4 negatively correlated with HAMD-17. In the slow-4 frequency band, the increased DC value in the cluster 1 brain area positively correlated with the BDI-21 score. We therefore speculate that the DC abnormality of the limbic system may be not only a compensatory manifestation of PD depression but also an important node of PD depression.

Fifth, this study found that abnormality in some brain areas is associated with PD, but not with depression.

It is well known that the putamen, globus pallidus, and thalamus are important components of the cortico-striatal-thalamo-cortical (CSTC) circuit. In this study, abnormal DC values in the CSTC circuit were observed in the PDND and PDD patients, suggesting that both PDND and PDD patients have CSTC loop neuronal function damage; this is also consistent with the currently known pathological mechanism of PD. More and more evidence show that the cerebellum has the traditional integrated motor function, which is mainly related to the motor symptoms of PD (Küper et al., 2011; Dirkx et al., 2017). In this study, PDD and PDND patients exhibited extensive changes of DC in the cerebellum in multiple frequency bands compared to HCs. We therefore speculate that the cerebellar region may be a compensatory manifestation of PDD.

We acknowledge some limitations of this study. First, the selection of the threshold for computing DC (*r* ≥ 0.25) in this study is subjective, although the threshold is consistent with previous studies (Buckner et al., 2009; Wang et al., 2018). However, Buckner et al. (2009) found that the selection of different thresholds for calculation will have a slight impact on the main results, and this study did not use other thresholds to calculate DC. Future studies on the DC changes in all frequency sub-bands are deserved. Second, this study is a cross-sectional study and did not follow dynamic brain function changes in PD patients with and without depression. Third, although the enrollment criteria of this study required that the participants needed to stop anti-PD medicines and antidepressants for at least 12 h (off period), it is still impossible to rule out the potential impact of long-term use of drugs on the experimental results. Fourth, due to the small sample size, we did not subdivide the degree of depression. This may affect the accuracy of the results. In future studies, large-sample, multi-center, and follow-up studies will be helpful to fully understand the neuroimaging mechanism of depression in PD.

## Conclusion

This study observed a wide change in DC at voxel level in different frequency bands in PD patients with and without depression. The brain function networks of PDD and PDND have different abnormal patterns in different frequency bands, and the corresponding DC abnormal brain areas have frequency specificity. The medial frontal gyrus, SMA, and limbic system may be the key hubs in the occurrence of depression in patients with PD. This study provides new ideas for further exploring the neuropathological mechanism of depression in PD.

## Data Availability Statement

The original contributions presented in the study are included in the article/supplementary material; further inquiries can be directed to the corresponding author/s.

## Ethics Statement

The studies involving human participants were reviewed and approved by This study was approved by the Ethics Committee of the Second Xiangya Hospital of Central South University. The patients/participants provided their written informed consent to participate in this study.

## Author Contributions

CT, HL, and JY contributed to the conception and design of the study. HL, JY, SC, QS, QL, LZ, JLi, ZM, TW, YZ, MW, and SL contributed to data collection. HL, JY, SC, Jliu, CW, and XZ contributed to data analysis. HL contributed to writing the manuscript. All authors read and approved the final revision.

## Conflict of Interest

The authors declare that the research was conducted in the absence of any commercial or financial relationships that could be construed as a potential conflict of interest.
